# Comparative Evaluation of Caudal Dexmedetomidine vs. Tramadol As Adjuvants to Bupivacaine for Intraoperative Hemodynamic Stability and Postoperative Analgesia in Pediatric Infraumbilical Surgeries

**DOI:** 10.7759/cureus.110691

**Published:** 2026-06-11

**Authors:** Naeem Abbas, Abdullah Bilal, Hafsa Tariq, Humaira Shahzadi, Muhammad Shahid, Adeel Riaz

**Affiliations:** 1 Anesthesiology and Critical Care, Sahiwal Medical College, Sahiwal, PAK; 2 Anesthesiology, Sahiwal Teaching Hospital, Sahiwal, PAK; 3 Radiation Oncology, Rutgers Cancer Institute, New Brunswick, USA

**Keywords:** bupivacaine, caudal analgesia, dexmedetomidine, infraumbilical surgery, pediatric anesthesia, regional anesthesia, tramadol

## Abstract

Background

Caudal epidural analgesia is a widely used and effective regional anesthetic technique for pediatric infraumbilical surgeries. Although bupivacaine provides reliable analgesia, its relatively short duration has prompted the use of adjuvants to prolong postoperative pain relief. Dexmedetomidine, a selective α2-adrenergic agonist, and tramadol, a centrally acting analgesic, are commonly employed caudal adjuvants; however, their comparative effects when combined with bupivacaine remain incompletely defined.

Methods

This prospective, randomized, double-blind study included 60 pediatric patients aged two to eight years undergoing elective infraumbilical surgeries under general anesthesia. All patients received a caudal block with 0.25% bupivacaine (1 mL/kg) and were randomized into two groups: Group D received dexmedetomidine 0.5 µg/kg (n = 30), and Group T received tramadol 1 mg/kg (n = 30). Hemodynamic parameters, including heart rate (HR) and mean arterial pressure (MAP), were recorded intraoperatively and at extubation. The primary outcome was time to first rescue analgesia, defined as the time from skin closure to a Face, Legs, Activity, Cry, Consolability (FLACC) score >4. Data were analyzed using appropriate parametric tests, with p < 0.05 considered statistically significant.

Results

Demographic characteristics and duration of surgery were comparable between the groups. Mean age was 4.19 ± 1.91 years in Group D and 4.55 ± 2.10 years in Group T (p = 0.49), while mean body weight was 14.15 ± 4.00 kg and 15.16 ± 4.24 kg, respectively (p = 0.35). The duration of postoperative analgesia was significantly longer in the dexmedetomidine group compared with the tramadol group (812.70 ± 46.15 minutes vs. 605.53 ± 45.12 minutes; p < 0.0001). HR and MAP decreased gradually in both groups following induction, with lower values observed in the dexmedetomidine group; however, differences in HR at all measured time points were not statistically significant. MAP after extubation was significantly lower in Group D compared with Group T (66.73 ± 5.96 mmHg vs. 70.17 ± 6.86 mmHg; p = 0.04). No episodes of bradycardia or hypotension requiring intervention occurred in either group.

Conclusion

Dexmedetomidine (0.5 µg/kg) as an adjuvant to caudal bupivacaine provided significantly prolonged postoperative analgesia compared with tramadol (1 mg/kg) in pediatric infraumbilical surgeries. Both agents maintained stable perioperative hemodynamics without clinically significant adverse effects; however, dexmedetomidine demonstrated better attenuation of hemodynamic responses, particularly after extubation. Low-dose dexmedetomidine appears to be an effective and safe caudal adjuvant in pediatric patients undergoing infraumbilical surgical procedures.

## Introduction

Effective postoperative analgesia following pediatric infraumbilical and lower abdominal surgeries is essential for enhanced recovery, reduced physiological stress, improved patient satisfaction, and prevention of long-term alterations in pain perception [1,2]. Procedures, such as inguinal hernia repair, circumcision, hypospadias repair, orchidopexy, and urethral surgeries, are commonly associated with moderate postoperative pain requiring reliable analgesia [3]. Caudal epidural anesthesia using long-acting local anesthetics like bupivacaine and ropivacaine is a safe, reliable, and frequently used technique in children because of its simplicity, high success rate, and effectiveness in providing intraoperative and postoperative analgesia while reducing anesthetic and opioid requirements, attenuating the stress response, and facilitating early recovery [4,5].

Bupivacaine and ropivacaine are amide local anesthetics that provide sensory and motor blockade by inhibiting sodium channel conduction [6]. Although ropivacaine has lower cardiotoxicity and causes less motor blockade, bupivacaine offers a faster onset, denser sensory block, and greater cost-effectiveness, making it the preferred option in many low-resource settings [7,8]. However, analgesia with bupivacaine alone usually lasts only four to six hours, which may be insufficient for procedures associated with prolonged postoperative pain. Consequently, several adjuvants, including opioids, α2-agonists, ketamine, midazolam, neostigmine, tramadol, and dexamethasone, have been investigated to prolong caudal analgesia with varying efficacy and side-effect profiles [9-15]. However, the search for an ideal adjuvant, one that prolongs analgesia without increasing adverse effects, continues.

Dexmedetomidine, a highly selective α2-adrenergic agonist with sedative, analgesic, and sympatholytic properties, prolongs caudal analgesia through spinal α2 receptor activation and systemic absorption without significant respiratory depression [16]. Previous studies have shown that dexmedetomidine enhances the duration and quality of caudal blocks when combined with bupivacaine or ropivacaine [17,18]. Tramadol, a centrally acting analgesic with weak μ-opioid agonism and inhibition of norepinephrine and serotonin reuptake, is another commonly used caudal adjuvant because of its relatively safe respiratory profile, though it may be associated with nausea, vomiting, pruritus, and less predictable analgesia [19-21]. While dexmedetomidine and tramadol have been compared previously with ropivacaine, limited data exist regarding their comparative efficacy with bupivacaine, particularly in low-resource settings. Furthermore, most studies used higher dexmedetomidine doses (1-2 μg/kg), which may increase bradycardia and sedation. Therefore, this study compared the analgesic efficacy, hemodynamic effects, block characteristics, and adverse-effect profiles of low-dose caudal dexmedetomidine (0.5 μg/kg) vs. tramadol (1 mg/kg) as adjuvants to 0.25% bupivacaine (1 mL/kg) in children aged two to 10 years undergoing infraumbilical surgeries, hypothesizing that dexmedetomidine would provide longer analgesia with comparable safety.

## Materials and methods

This single-center, prospective, randomized, double-blind controlled trial was conducted over 12 months at the Department of Anesthesiology, Sahiwal Teaching Hospital, after approval from the Institutional Ethical Review Committee and in accordance with the Declaration of Helsinki. Written informed consent was obtained from parents or legal guardians of all participants. Pediatric patients aged two to eight years with American Society of Anesthesiologists (ASA) physical status I-II undergoing elective infraumbilical surgery under general anesthesia with planned single-shot caudal analgesia were included. Exclusion criteria included allergy to study drugs, infection, sepsis, coagulopathy (international normalized ratio (INR) > 1.5, prothrombin time (PT) > 15 seconds, or platelet count < 100 × 10⁹/L), neurological or spinal abnormalities, significant cardiopulmonary disease, developmental delay affecting pain assessment, and emergency surgery. Sixty patients were randomized equally using a sealed-envelope lottery method into Group D, receiving 0.25% bupivacaine 1 mL/kg with dexmedetomidine 0.5 µg/kg, or Group T, receiving 0.25% bupivacaine 1 mL/kg with tramadol 1 mg/kg. Study solutions were prepared in identical syringes by an anesthesiologist not involved in patient management or assessment. Consequently, the anesthesiologist performing the caudal block, the intraoperative care team, and the postoperative assessors - including nurses and research staff - remained blinded to group assignments. 

After standard fasting and monitoring (ECG, non-invasive blood pressure, pulse oximetry, and end-tidal CO₂), anesthesia was induced with propofol 2.5 mg/kg IV and atracurium 0.5 mg/kg, followed by tracheal intubation and maintenance with sevoflurane in 50% oxygen-air mixture. Caudal block was performed in the left lateral position using anatomical landmarks under strict aseptic precautions with a 22-gauge short bevel needle. Correct placement was confirmed by negative aspiration and whoosh test before slow injection of the study solution [22]. Patients with suspected intravascular or intrathecal injection were excluded. Hemodynamic parameters, including heart rate (HR), mean arterial pressure (MAP), and SpO₂ were recorded at baseline, after caudal injection (T0), at five, 10, 15, 30, 45, and 60 minutes intraoperatively, and at extubation. Bradycardia, tachycardia, and hypotension were defined as >20% change from baseline and managed according to a predefined protocol. Bradycardia was treated with atropine 0.02 mg/kg body weight intravenously (IV). Hypotension was managed with fluid resuscitation and, when clinically indicated, IV epinephrine 0.5 μg/kg body weight. Tachycardia was managed by identifying and correcting underlying causes, including fluid resuscitation for hypovolemia and appropriate analgesia for pain; persistent supraventricular tachycardia was treated with IV adenosine 0.1 mg/kg body weight. Rescue intraoperative analgesia, surgical duration, and postoperative recovery were documented. 

**Figure 1:**
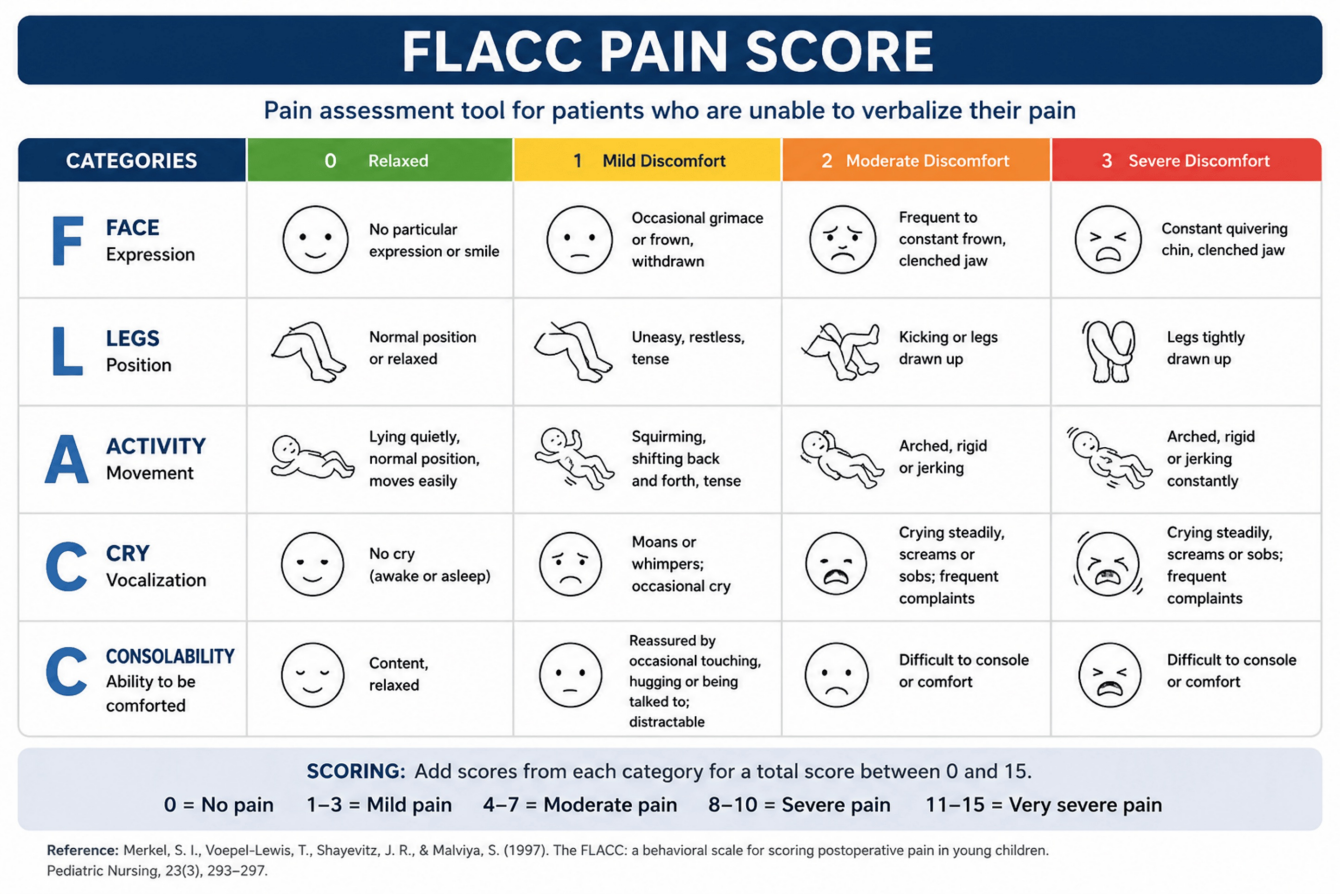
Face, Legs, Activity, Cry, Consolability (FLACC) Behavioral Pain Assessment Scale Reference: [23]

The primary outcome was duration of analgesia, defined as the time from skin closure to first rescue analgesia when the Face, Legs, Activity, Cry, Consolability (FLACC) score exceeded 4. Secondary outcomes included HR and MAP changes. Postoperative pain was assessed using the FLACC pain scale, as shown in Figure 1 below, at one, two, four, six, eight, 12, and 24 hours.

Rescue analgesia consisted of morphine 0.05 mg/kg, with additional doses and paracetamol 15 mg/kg if required. Sample size was calculated using the OpenEpi calculator (Dean AG, Sullivan KM, Soe MM, Emory University, Atlanta, GA) for comparing two means from the reference study conducted by Gupta et al. [24]. Based on a 95% confidence interval, a study power of 80%, a mean duration of analgesia of 780.29 ± 71.21 minutes in the dexmedetomidine group, and 654.20 ± 78.38 minutes in the tramadol group, the required sample size was calculated to be 12 patients, with six patients in each group, using the following formula:

$n = \frac{2(Z_{\alpha/2} + Z_{\beta})^{2}\sigma^{2}}{(\mu_{1} - \mu_{2})^{2}}$

where n = sample size per group; Zα/2 = standard normal deviate for the chosen confidence level (1.96 for 95% confidence); Zβ = standard normal deviate for the desired power (0.84 for 80% power); σ² = pooled variance; μ₁ - μ₂ = expected difference between the two group means.

A total of 71 patients were assessed, but 11 patients were excluded based on exclusion criteria. Seven had surgery planned for emergency issues, two had sepsis, one had a known neurologic condition, and consent was revoked after the procedure for one patient. A total of 60 patients (30 per group) were enrolled to improve statistical power and account for dropouts. Data were analyzed using SPSS version 26 (IBM SPSS Statistics for Windows, IBM Corp., Armonk, NY). Continuous variables were assessed for normality using the Shapiro-Wilk test and analyzed with an independent t-test, while categorical variables were compared using chi-square or Fisher’s exact test. Repeated hemodynamic measurements were analyzed using repeated measures ANOVA with Greenhouse-Geisser correction and Bonferroni-adjusted post-hoc testing. A p-value of <0.05 was considered statistically significant.

## Results

A total of 60 pediatric patients aged two to eight years undergoing elective infraumbilical surgeries, including inguinal hernia repair (the most common procedure performed), appendectomy, orchidopexy, circumcision, and hydrocele repair, were enrolled and randomly allocated into two equal groups: Group D received caudal bupivacaine with dexmedetomidine (n = 30), while Group T received caudal bupivacaine with tramadol (n = 30). Demographic characteristics were comparable between the groups, as shown in Table 1. 

**Table 1:** Demographics, drugs, type of surgery, and duration of surgery in each group

Parameter	Group D (n = 30)	Group T (n = 30)
Drugs	Dexmedetomidine with bupivacaine	Tramadol with bupivacaine
Sex (male:female)	27:3	27:3
Age (years)	4.19 ± 1.912	4.55 ± 2.106
Weight (kilograms)	14.15 ± 4	15.16 ± 4.2
Type of surgery	Inguinal hernia (n = 20), appendectomy (n = 6), circumcision (n = 4), orchidopexy (n = 0)	Inguinal hernia (n = 19), appendectomy, (n = 5), circumcision (n = 5), orchidopexy (n = 1)
Duration of surgery (minutes)	38.5 ± 8.4	39.2 ± 7.6

The mean age was 4.19 ± 1.91 years in Group D and 4.55 ± 2.10 years in Group T (p = 0.49). Mean body weight was 14.15 ± 4.00 kg in Group D and 15.16 ± 4.24 kg in Group T (p = 0.35). Baseline HR before induction was also similar between the groups, measuring 103.70 ± 10.60 beats/min in Group D and 103.20 ± 10.62 beats/minute in Group T (p = 0.86). The duration of surgery was also similar, 38.5 ± 8.4 minutes in Group D vs. 39.2 ± 7.6 minutes in Group T. 

The primary outcome, time to rescue analgesia, was significantly prolonged in the dexmedetomidine group compared with the tramadol group. Mean time to rescue analgesia was 812.70 ± 46.15 minutes in Group D vs. 605.53 ± 45.12 minutes in Group T, and this difference was highly statistically significant (p < 0.0001). Primary and secondary outcomes are shown in Table 2.

**Table 2:** Primary and secondary outcomes for respective groups HR, heart rate; MAP, mean arterial pressure

Parameter	Group D (n = 30)	Group T (n = 30)	p-value	t-value
Time to rescue analgesia	812.70 ± 46.15	605.53 ± 45.12	<0.0001*	17.581
HR before induction	103.7 ± 10.6	103.2 ± 10.6	0.86	0.183
HR after 5 minutes	100.27 ± 9.5	98.3 ± 11.9	0.48	0.709
HR after 10 minutes	94.93 ± 8.6	95.07 ± 12.1	0.96	-0.052
HR after 15 minutes	89.63 ± 18	92.37 ± 11.2	0.48	-0.708
HR after 30 minutes	89.87 ± 10.2	92.1 ± 12.7	0.46	-0.750
HR after extubation	91.37 ± 10.07	96.07 ± 11.33	0.09	-1.698
MAP before induction	69.33 ± 8.31	71.52 ± 8.37	0.31	-1.017
MAP after 5 minutes	67.53 ± 6.21	70.48 ± 6.70	0.08	-1.769
MAP after 10 minutes	65.53 ± 7.40	68.80 ± 7.62	0.10	-1.686
MAP after 15 minutes	66.67 ± 6.81	69.37 ± 7.53	0.15	-1.457
MAP after 30 minutes	66.90 ± 5.32	69.44 ± 6.76	0.11	-1.617
MAP after extubation	66.73 ± 5.96	70.17 ± 6.86	0.04*	-2.073

Following induction, HR gradually decreased in both groups. At five minutes, mean HR was 100.27 ± 9.55 beats/minute in Group D and 98.30 ± 11.89 beats/minute in Group T (p = 0.48). At 10 minutes, HR decreased to 94.93 ± 8.61 beats/minute and 95.07 ± 12.07 beats/minute, respectively (p = 0.96). At 15 minutes, HR was 89.63 ± 18.03 beats/minute in Group D and 92.37 ± 11.17 beats/minute in Group T (p = 0.48). At 30 minutes, corresponding values were 89.87 ± 10.19 beats/minute and 92.10 ± 12.74 beats/minute, respectively (p = 0.46). Following extubation, mean HR was 91.37 ± 10.07 beats/minute in Group D compared with 96.07 ± 11.33 beats/minute in Group T (p = 0.09). None of these differences reached statistical significance.

MAP before induction was 69.33 ± 8.31 mmHg in Group D and 71.52 ± 8.37 mmHg in Group T (p = 0.31). At five minutes after induction, MAP was 67.53 ± 6.21 mmHg in Group D and 70.48 ± 6.70 mmHg in Group T (p = 0.08). At 10 minutes, MAP values were 65.53 ± 7.40 mmHg and 68.80 ± 7.62 mmHg, respectively (p = 0.10). At 15 minutes, MAP was 66.67 ± 6.81 mmHg in Group D and 69.37 ± 7.53 mmHg in Group T (p = 0.15). At 30 minutes, MAP values were 66.90 ± 5.32 mmHg and 69.44 ± 6.76 mmHg, respectively (p = 0.11). Following extubation, MAP was 66.73 ± 5.96 mmHg in Group D compared with 70.17 ± 6.86 mmHg in Group T, and this difference was statistically significant (p = 0.04). No episodes of bradycardia or hypotension requiring intervention were reported in either group. 

## Discussion

The present study compared the analgesic efficacy, hemodynamic effects, and safety profile of dexmedetomidine (0.5 µg/kg) and tramadol (1 mg/kg) as adjuvants to caudal bupivacaine in pediatric patients undergoing infraumbilical surgeries. The findings demonstrated that both adjuvants provided effective caudal analgesia with stable perioperative hemodynamics; however, dexmedetomidine produced a significantly longer duration of postoperative analgesia and better attenuation of hemodynamic responses compared with tramadol, without clinically significant adverse effects. 

The demographic characteristics and duration of surgery were comparable between the two groups, minimizing confounding variables and allowing meaningful comparison of outcomes. The significantly prolonged duration of analgesia observed in the dexmedetomidine group (812.70 ± 46.15 minutes) compared with the tramadol group (605.53 ± 45.12 minutes; p < 0.0001) is consistent with the pharmacological profile of α2-adrenergic agonists, which inhibit nociceptive transmission at spinal and supraspinal levels. Similar findings have been reported in previous studies where dexmedetomidine prolonged caudal analgesia when combined with local anesthetics compared with opioids or local anesthetics alone [17,18]. The prolonged analgesic duration observed in the present study suggests that dexmedetomidine may reduce postoperative analgesic requirements and improve postoperative comfort in pediatric patients. 

HR and MAP gradually decreased in both groups following induction. Although values were consistently lower in the dexmedetomidine group, the differences did not reach statistical significance. Despite these trends, differences in HR between groups at five, 10, 15, and 30 minutes intraoperatively and after extubation were not statistically significant. Similarly, MAP differences during the intraoperative period did not reach statistical significance, although MAP after extubation was significantly lower in the dexmedetomidine group (p = 0.04). Importantly, all hemodynamic values remained within clinically acceptable ranges, and no patient required atropine for bradycardia or vasopressor support for hypotension. These findings support the hemodynamic safety of low-dose dexmedetomidine and are consistent with previous pediatric studies demonstrating that dexmedetomidine attenuates perioperative sympathetic responses while preserving cardiovascular stability. 

Higher dexmedetomidine doses (1-2 µg/kg) have previously been associated with bradycardia, hypotension, and excessive sedation [24]. By utilizing a lower dose of 0.5 µg/kg, the present study demonstrated that effective prolongation of analgesia and favorable hemodynamic modulation can be achieved without increasing adverse effects, highlighting the importance of dose optimization in pediatric regional anesthesia. In contrast, tramadol, although widely used because of its relatively safe respiratory profile and availability, provided a shorter duration of analgesia and less pronounced attenuation of hemodynamic responses. No opioid-related adverse effects, such as respiratory depression, were observed in either group. The absence of major adverse effects in both groups in the present study supports the overall safety of both agents; however, dexmedetomidine appears to offer a more favorable balance between analgesic efficacy and physiological stability. 

The findings of this study should be interpreted in light of certain limitations. The study was conducted at a single center with a relatively small sample size, which may limit generalizability. Sedation scores and postoperative adverse effects, such as nausea and vomiting, were not formally assessed, which may have provided additional information regarding recovery characteristics and tolerability of the study drugs. Larger multicenter studies with comprehensive postoperative recovery assessment may further clarify the comparative efficacy and safety profiles of dexmedetomidine and tramadol as caudal adjuvants in pediatric patients. 

## Conclusions

Dexmedetomidine (0.5 µg/kg) as an adjuvant to caudal bupivacaine provided significantly longer postoperative analgesia compared with tramadol (1 mg/kg) in pediatric patients undergoing infraumbilical surgeries. Both agents maintained stable perioperative hemodynamics without clinically significant adverse effects; however, dexmedetomidine demonstrated better attenuation of hemodynamic responses, particularly following extubation. The findings suggest that low-dose dexmedetomidine is an effective and safe caudal adjuvant for prolonging postoperative analgesia while maintaining hemodynamic stability in children undergoing infraumbilical surgical procedures. 
